# FGA influences invasion and metastasis of hepatocellular carcinoma through the PI3K/AKT pathway

**DOI:** 10.18632/aging.206011

**Published:** 2024-07-09

**Authors:** Xi Han, Zefeng Liu, Mengying Cui, Jie Lin, Yongzhi Li, Hanjiao Qin, Jiyao Sheng, Xuewen Zhang

**Affiliations:** 1 Department of Hepatobiliary and Pancreatic Surgery, Second Hospital of Jilin University, Changchun, Jilin 130041, China; 2 Department of Radiotherapy, Second Hospital of Jilin University, Changchun, Jilin 130041, China

**Keywords:** FGA, hepatocellular carcinoma, PI3K, AKT, metastasis

## Abstract

Fibrinogen is an important plasma protein composed of three polypeptide chains, fibrinogen alpha (FGA), beta, and gamma. Apart from being an inflammation regulator, fibrinogen also plays a role in tumor progression. Liver cancer usually has a poor prognosis, with chronic hepatitis being the main cause of liver cirrhosis and hepatocellular carcinoma (HCC). FGA serves as a serological marker for chronic hepatitis, but its relationship with liver cancer remains unclear. Through bioinformatics analysis and agarose gel electrophoresis, we found that FGA was downregulated in HCC and correlated with tumor stage and grade. By constructing both *FGA* gene knockout and overexpression cell models, we demonstrated that overexpressing *FGA* inhibited migration and invasion of liver cancer cells through Transwell migration/invasion and wound healing assays. Western blotting experiments showed that *FGA* overexpression increased the expression of the epithelial-mesenchymal transition marker protein E-cadherin while decreasing N-cadherin and slug protein expression. In addition, *FGA* knockout activated the PI3K/AKT pathway. In a mouse model of metastatic tumors, overexpression of FGA restricted the spread of tumor cells. In conclusion, FGA exhibits an inhibitory effect on tumor metastasis, providing new insights for the treatment of advanced HCC metastatic tumors.

## INTRODUCTION

Liver cancer is the sixth most common cancer worldwide and the third leading cause of cancer-related deaths globally [1]. Hepatocellular carcinoma (HCC), which is the most common type of liver cancer, accounts for 75%–85% of cases [2]. HCC typically presents with few symptoms. Although detection methods have significantly improved, there has not been a substantial increase in survival rates, especially for patients with intrahepatic and extrahepatic metastases [3, 4]. Given that invasion and metastasis are significant factors affecting the prognosis of cancer patients, it is crucial to explore the mechanisms of HCC invasion and metastasis. HCC typically presents with few symptoms. Although detection methods have significantly improved, there has not been a substantial increase in survival rates, especially for patients with intrahepatic and extrahepatic metastases [5–7]. Research has shown that fibrinogen plays a role in the metastatic mechanism of solid tumors and is an important factor influencing the migration ability of tumor cells [8–11].

In most cases, HCC arises from chronic liver disease. Patients sequentially go through hepatitis, fibrosis, cirrhosis, and ultimately develop HCC [12–14]. Thus, liver cancer is closely linked to inflammation, and prolonged chronic hepatitis is a major cause of HCC and cirrhosis [15]. Neutrophils, macrophages, and other cells are recruited to the liver, producing cytokines and chemokines, promoting the progression of liver fibrosis [16, 17]. In a 15-year follow-up study, it was found that tumor-associated neutrophils (TANs) recruited inflammatory cells, promoting the progression of HCC. The levels of the cytokines expressed by TANs correlated with microvascular invasion, tumor differentiation, and staging. Moreover, patients expressing high levels of CCL2 and CCL17 had shorter survival times [18]. In inflammation-induced HCC, the overexpression of KLF7 promoted the progression and metastasis of HCC [19].

Multiple lines of evidence support the vital role of fibrinogen and its degradation products in regulating inflammatory responses in various target tissues [20]. They were found to promote tumor cell metastasis by inhibiting immune cell activity [21]. FGA has been shown to be a serological marker for chronic hepatitis, in which expression of the 5.9 kDa fragment of the FGA C-chain was inhibited [22]. In a deep whole-genome analysis of Chinese HCC published in the journal Nature, six candidate coding driver factors, including FGA, were identified in the Chinese liver cancer landscape, which play a certain role in regulating the progression and metastasis of HCC [23]. In the HepG2 cell line, inflammation-associated TGF-β inhibited fibrinogen induction produced by IL-6 and reduced fibrinogen synthesis, which did not rule out the possibility that TGF-β regulated the synthesis of α-fibrinogen at the transcriptional level [24]. *In vivo* experiments by Steinbrecher and others found that the number of colon adenoma formations was significantly reduced in fibrinogen-deficient mice, confirming that fibrinogen mediated local inflammation through the leukocyte integrin α(M)β(2), thereby creating an inflammatory microenvironment that may be conducive to tumor progression [25]. In addition, fibrinogen-β was found to interact with the hepatitis C virus (HCV) core, interfering with the immune response [26].

In this study, based on an analysis of the TCGA database, it is shown that FGA had low expression in HCC and is related to the staging and grading of HCC. To study the impact of FGA on HCC cell invasion and metastasis, we created *FGA* knockout and overexpression cell models in the HCC cell line by using the CRISPR/Cas9 genome editing technique. We then investigated the effect of FGA on tumor development and explored potential signaling regulatory pathways.

## RESULTS

### In comparison to normal tissue, the expression level of FGA is reduced in human liver cancer

**Figure 1:**
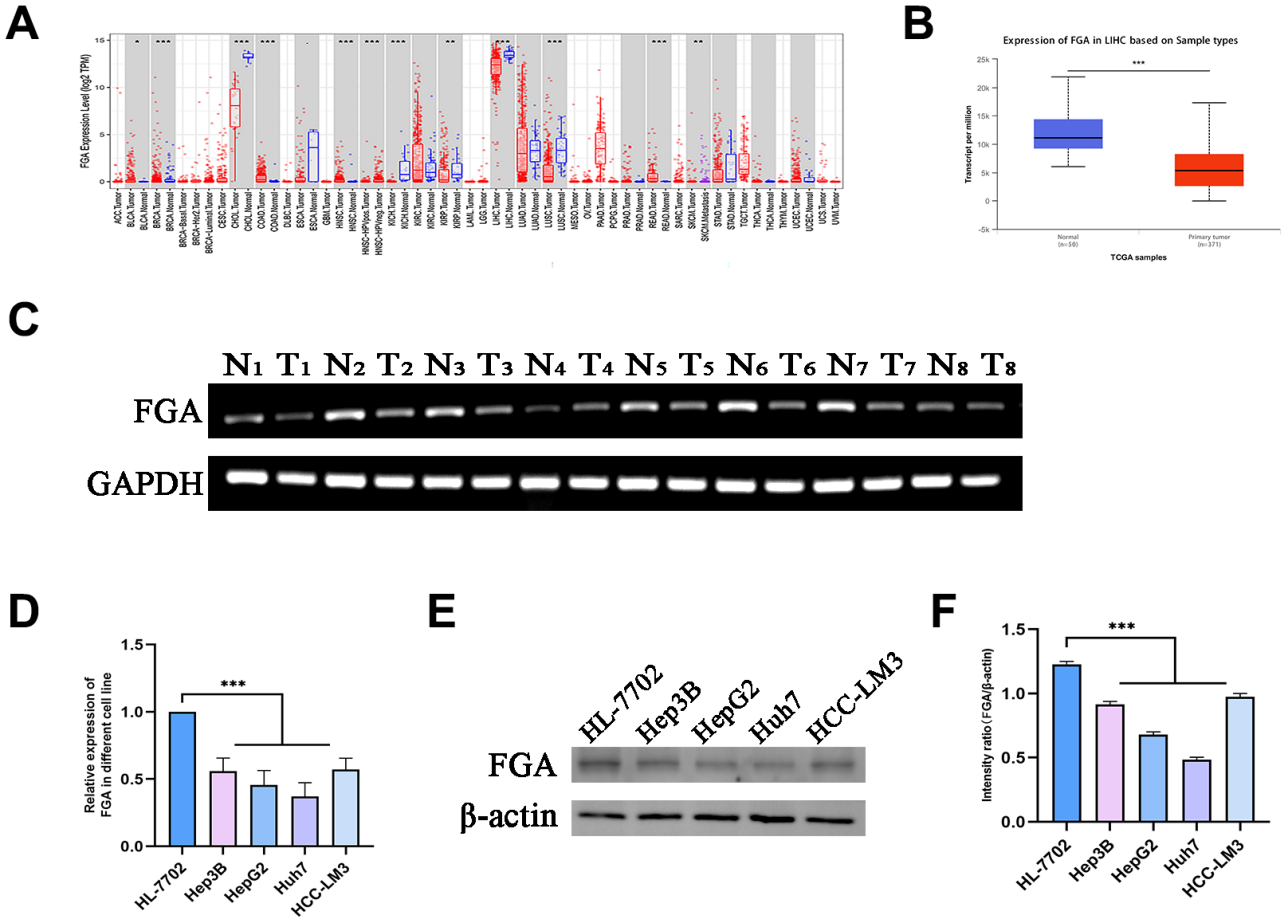
***FGA* is underexpressed in liver cancer tissue.** (**A**) Analysis of differential *FGA* expression between liver cancer and adjacent non-cancerous tissues using TCGA RNA-seq data through the TIMER database. (**B**) Evaluation of *FGA* expression levels in hepatocellular carcinoma using UNCLAN data. (**C**) Detection of *FGA* expression in eight cases of adjacent non-cancerous tissue and eight cases of liver cancer tissue through agarose gel electrophoresis assay. (**D**) Detection of FGA mRNA expression in normal liver cell line HL-7702 and different liver cancer cell lines using RT-qPCR. (**E**) Detection of FGA protein expression in normal liver cell line HL-7702 and different liver cancer cell lines using Western blot, and its quantitative analysis. (**F**) (N: normal tissue, T: tumor tissue; two-tailed Student’s t test; mean ± SD, n = 3; *P < 0.05, **P < 0.01, ***P < 0.001, compared to the normal group).

To investigate the role of FGA in HCC, through the TIMER database, we analyzed TCGA RNA-seq data and assessed the transcript levels of *FGA* in various human tumors. We observed that *FGA* expression was lower in liver carcinoma tissue. In addition, *FGA* was found to be under-expressed in bile duct carcinoma, lung squamous cell carcinoma, and kidney chromophobe tissue, while it was overexpressed in colon adenocarcinoma and rectal adenocarcinoma, indicating abnormal FGA expression across multiple types of tumors (Figure 1A). Furthermore, using UNCLAN data to evaluate FGA expression levels in hepatocellular carcinoma, we observed that FGA expression was significantly decreased in HCC compared to normal tissue (P < 0.001) (Figure 1B). Additionally, we collected 8 sets of clinical specimens, each set including cancer and adjacent tissue. We extracted RNA from all tissues, obtained PCR products, and conducted agarose gel electrophoresis experiments, yielding the same conclusions (Figure 1C). Meanwhile, we compared the FGA mRNA (Figure 1D) and protein expression (Figure 1E, 1F) between normal liver cell lines and liver cancer cell lines using RT-qPCR and Western blot methods, and found that the expression level of FGA in liver cancer cell lines was generally lower than that in normal liver cell lines, further confirming the differential expression of FGA. In summary, these results suggested that FGA expression is lower in hepatocellular carcinoma tissue compared to normal liver tissue.

### Clinical and pathological characteristics associated with *FGA* in HCC patients

**Figure 2:**
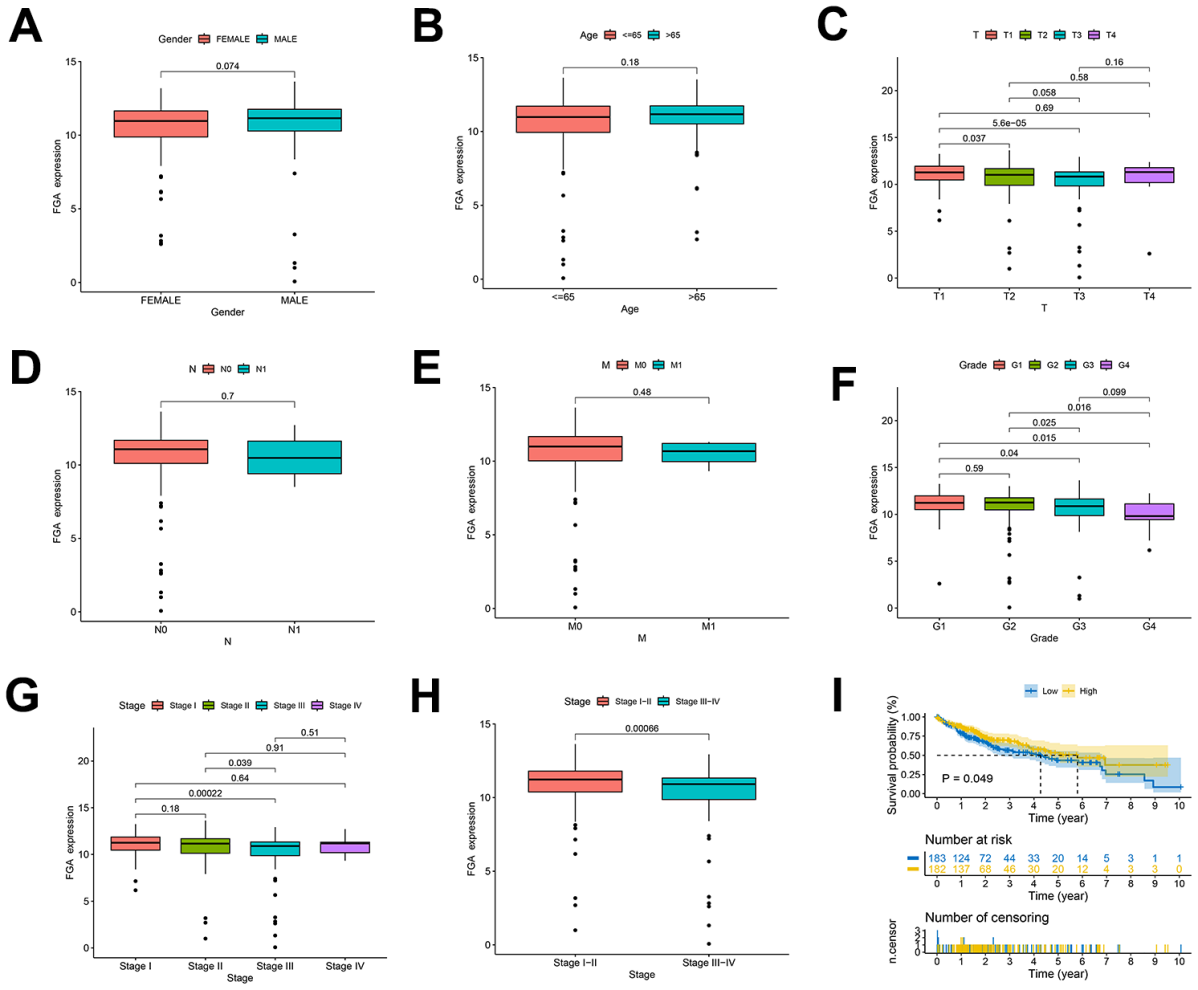
**Clinical and pathological characteristics of FGA in HCC patients.** (**A**–**H**) Evaluation of *FGA* expression levels in HCC patients using TCGA data, showing the relationship between *FGA* expression and patient gender, age, depth of infiltration, lymph node metastasis, distant metastasis, grade, TNM stage, and overall survival. (**I**) Kaplan–Meier curves displaying HCC patients with high expression of *FGA* gain favorable overall survival. (T: tumor, N: lymph node, M: metastasis; two-tailed Student’s t test; mean ± SD; *P < 0.05, **P < 0.01, ***P < 0.001).

We conducted a study using TCGA data to investigate *FGA* characteristics in clinical and pathological aspects. These characteristics included gender, age, depth of infiltration, grade, and TNM stage, lymph node metastasis, distant metastasis. *FGA* mRNA expression showed no significant correlation with factors such as age (P = 0.18), gender (P = 0.074), lymph node metastasis (P = 0.7), or distant metastasis (P = 0.48) in HCC patients (Figure 2A, 2B, 2D, 2E). However, *FGA* mRNA expression was significantly correlated with depth of infiltration, grade, and TNM stage (Figure 2C, 2F–2H). There were differences in *FGA* mRNA expression among different stages of liver cancer, and when we divided liver cancer into early (I and II) and late (III and IV) stages, *FGA* mRNA expression was significantly lower in late-stage liver cancer, indicating that *FGA* undergoes significant changes with the progression of liver cancer (P = 0.00066) (Figure 2H). Furthermore, the analysis of the prognostic value of *FGA* mRNA expression in HCC revealed that lower levels of *FGA* mRNA expression were significantly associated with a shorter overall survival period (Figure 2I).

### FGA inhibits the migration and invasion of HCC cells

**Figure 3:**
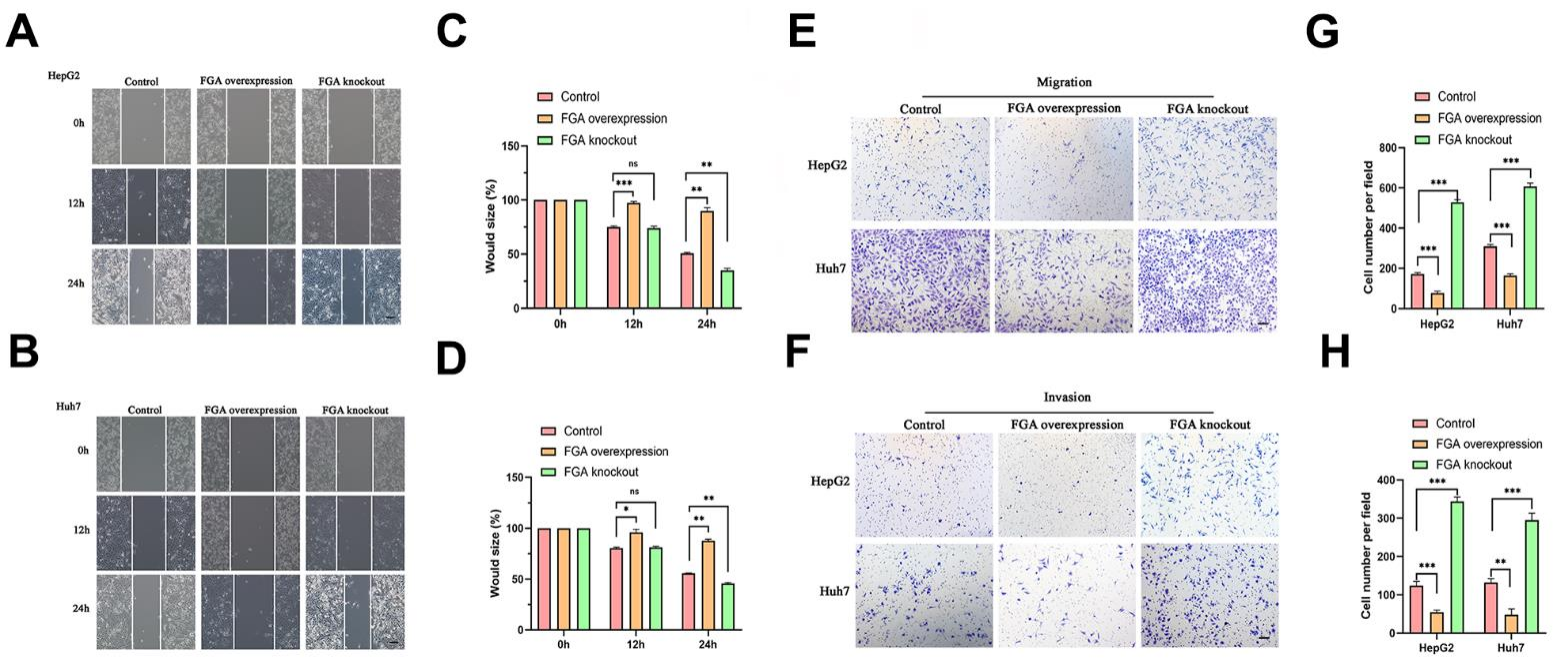
**The impact of FGA on HCC cell migration and invasion.** (**A**, **B**) Representative images were obtained from the wound healing assay, and the corresponding quantitative analysis of the results of the HepG2 (**C**) and Huh7 (**D**) wound healing assays demonstrates the effects of *FGA* overexpression and knockout in cell migration. (**E**, **F**) Representative images were acquired from the Transwell assay, and the corresponding quantitative analysis of the results of migration (**G**) and invasion (**H**) in the Transwell assay illustrate the impact of *FGA* overexpression and knockout on cell migration. (mean ± SD, n = 3; *P < 0.05, **P < 0.01, ***P < 0.001, compared with the control group. Scale: 100 μm).

To investigate the relationship between FGA and the migratory and invasive behavior of HCC, we explored the effects of FGA on two HCC cell lines, HepG2 and Huh7, using a wound healing assay (Figure 3A, 3B). Each cell line was divided into three groups. When *FGA* was overexpressed, there was no significant reduction in the wound area (Figure 3C, 3D). However, when we compared the data from the three groups, we found that the wound area was significantly reduced in the *FGA* knockout group (P < 0.01). Because the wound healing assay cannot exclude the influence of cell proliferation factors, we also performed a Transwell migration/invasion assay (Figure 3E, 3F). By counting the number of cells in the lower chamber, we found that *FGA* overexpression significantly inhibited cell migration (Figure 3G). In the Transwell invasion assay, *FGA* knockout significantly promoted cell invasion efficiency (Figure 3H). Therefore, low expression of *FGA* can promote the malignant behavior of HCC.

### FGA is involved in tumor cell migration through the PI3K/AKT pathway

**Figure 4:**
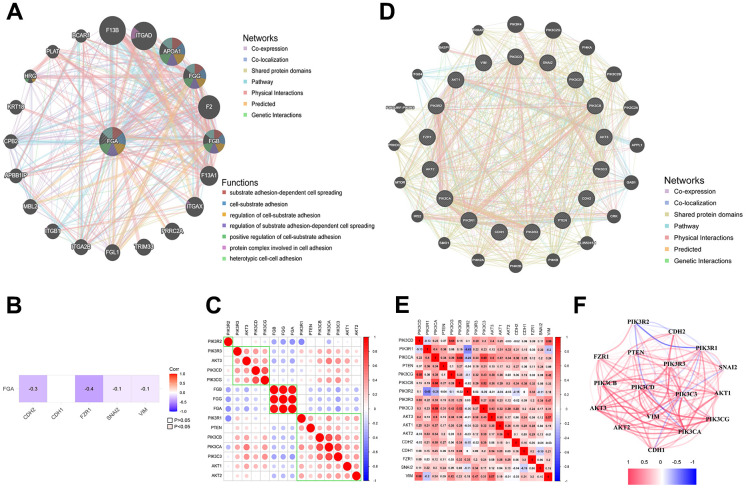
**FGA is involved in tumor cell migration through the PI3K/AKT pathway.** (**A**) FGA protein interaction network. (**B**) The relationship between FGA and EMT marker molecules. (**C**) The connection between FGA and PI3K/AKT pathway-related molecules in liver cancer. (**D**) Protein interaction network of EMT marker molecules with PI3K/AKT pathway-related molecules. (**E**) The correlation between EMT marker molecules and PI3K/AKT pathway-related molecules in liver cancer. (**F**) Network diagram showing the connections between EMT marker molecules and PI3K/AKT pathway-related molecules, with red indicating positive correlations and blue indicating negative correlations.

In order to explore the pathway through which FGA is involved in HCC metastasis, we analyzed the GeneMANIA database and found that FGA is associated with various cell signaling pathways, including cell-matrix adhesion and substrate adhesion-dependent cell spreading (Figure 4A). Abnormal expression of cell adhesion factors can confer tumor cells with migratory and invasive capabilities [27, 28]. The epithelial-mesenchymal transition (EMT) has a close relationship with malignant tumors, during which tumor cells gradually lose their epithelial cell-cell adhesion, promoting cancer progression [29]. Therefore, we analyzed the correlation between FGA and EMT-related marker protein (E-cadherin, N-cadherin, slug) in the TCGA database and found that FGA was negatively correlated with all of them (Figure 4B). It is reported that the PI3K/AKT pathway plays a crucial role in the progression of HCC [30, 31]. Through the analysis of the TCGA database, we found there was an association between FGA and the PI3K/AKT signaling pathway (Figure 4C). Using the GeneMANIA database and TCGA database, we identified the interaction and correlation between PI3K/AKT and E-cadherin, N-cadherin, and slug (Figure 4D–4F).

### FGA inhibits the epithelial-mesenchymal transition through the PI3K/AKT pathway

**Figure 5:**
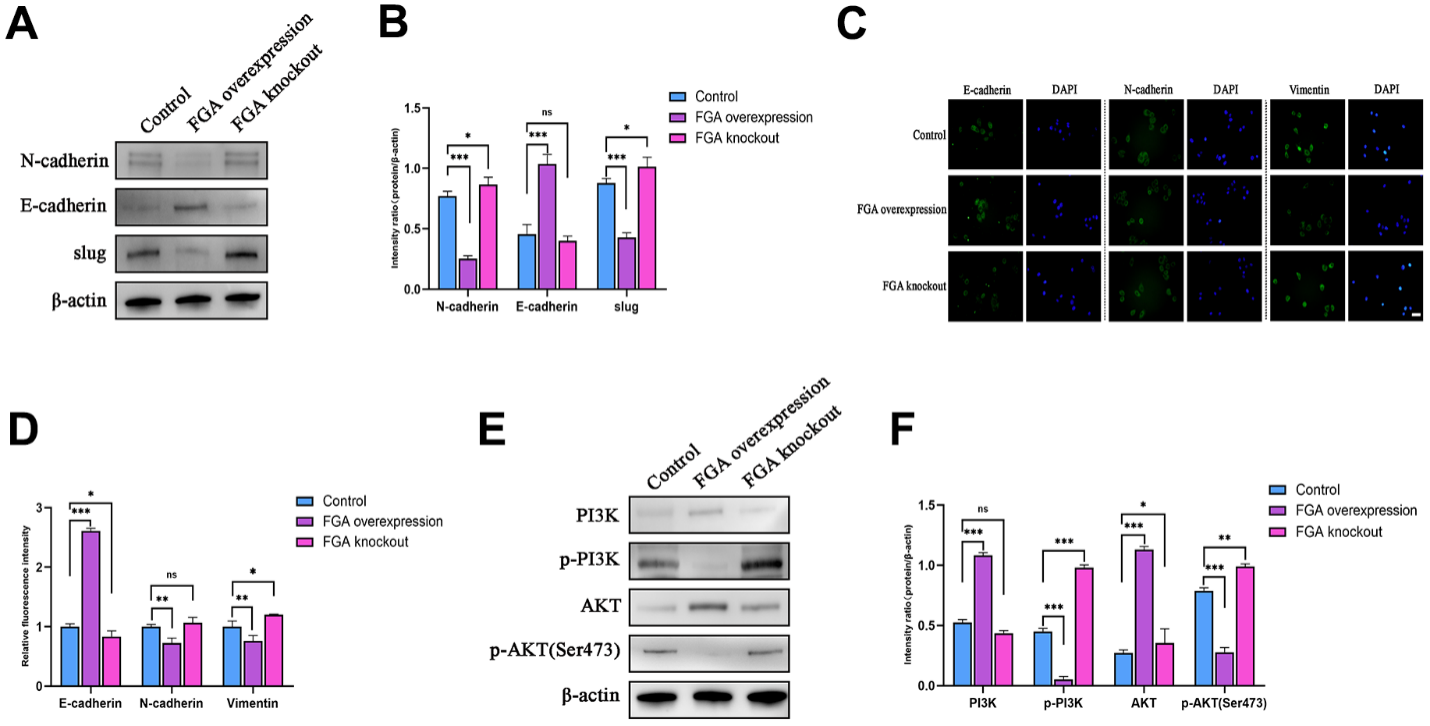
**FGA inhibits the epithelial-mesenchymal transition (EMT) through the PI3K/AKT signaling pathway.** (**A**) Western blot analysis of the expression levels of key EMT proteins (E-cadherin, N-cadherin, slug) in the control group, *FGA* knockout group and *FGA* overexpressed group, and their quantitative analysis (**B**). (**C**) Immunofluorescence staining of the expression levels of E-cadherin, N-cadherin, and vimentin proteins in the control group, *FGA* knockout group and *FGA* overexpressed group, and their quantitative analysis (**D**). (**E**, **F**) Western blot analysis of the expression levels of proteins related to the PI3K/AKT signaling pathway in the control group, *FGA* knockout group and *FGA* overexpressed group, and their quantitative analysis. (mean ± SD, n = 3; *P < 0.05, **P < 0.01, ***P < 0.001, compared with the control group. Scale: 20 μm)

To clarify whether FGA inhibits tumor metastasis by suppressing the EMT, we detected the expression of EMT-related marker proteins. As shown in Figure 5A, 5B, E-cadherin expression increased with *FGA* overexpression, while the expression of N-cadherin and slug proteins decreased, and when *FGA* was knocked out, the expression of N-cadherin and slug was slightly increased, indicating that changes in *FGA* expression were able to affect the expression of EMT-related proteins in some way. Immunofluorescence also confirmed this result (Figure 5C, 5D). The above results indicate that FGA negatively regulated the hallmark event of HCC cell invasion and metastasis, which is the EMT. Next, we studied the effects of *FGA* overexpression and *FGA* knockout on the expression of PI3K and AKT proteins. As depicted in Figure 5E, 5F, with *FGA* overexpression, the expression of p-PI3K and p-AKT (Ser473) decreased. In contrast, when *FGA* was knocked out, the expression both increased, indicating that *FGA* knockout activated the PI3K/AKT signaling pathway, promoting tumor metastasis.

### FGA inhibition of tumor metastasis *in vivo* experiment

**Figure 6:**
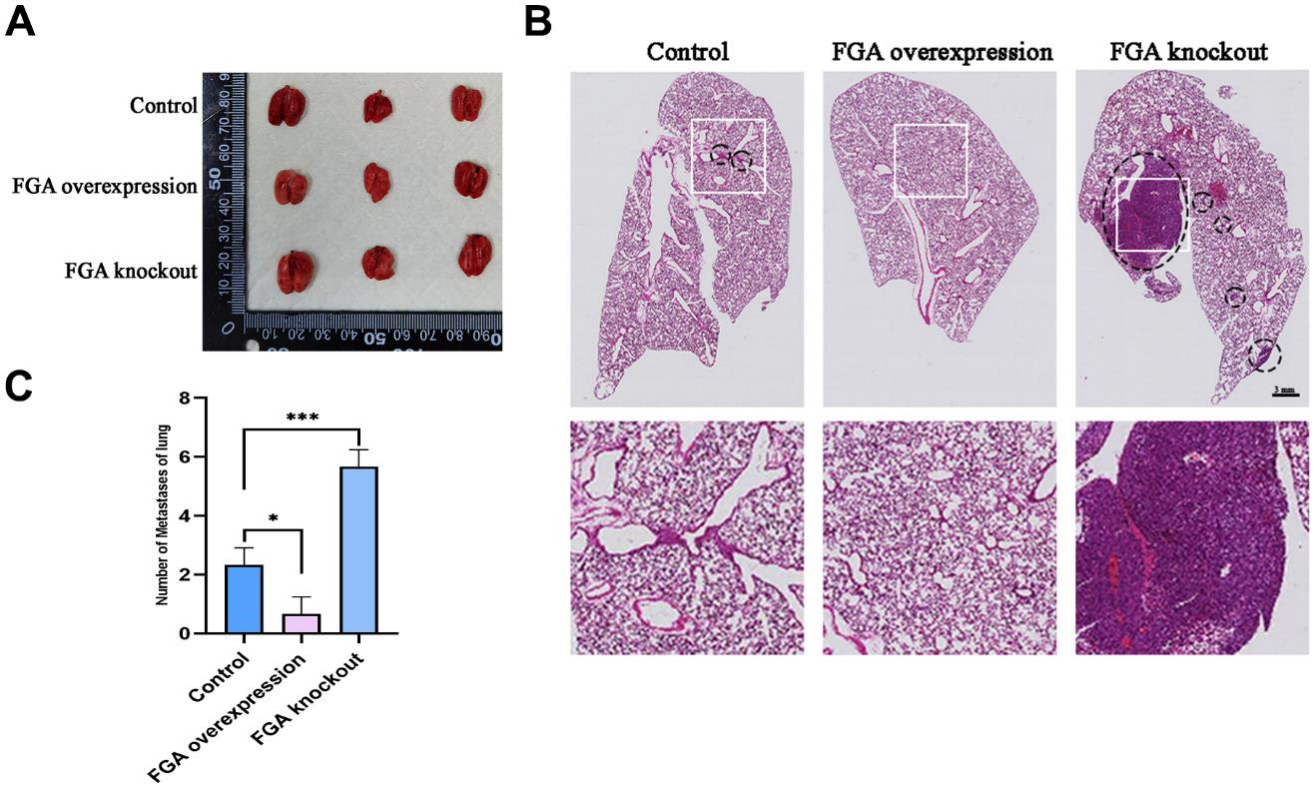
**The impact of FGA on HCC metastasis *in vivo*.** (**A**) Representative photographs of the lungs. (**B**) Representative images of lung biopsy, stained with H&E. Within the circle are metastatic cancer lesions. (**C**) Numbers of metastases of lung in each group. (mean ± SD, n = 3; *P < 0.05, **P < 0.01, ***P < 0.001, compared with the control group).

To verify the effect of FGA on tumor metastasis *in vivo*, the wild-type, FGA-overexpression and FGA-knockout HepG2 cells were implanted *in situ* in the livers of 4-week-old immunodeficient BALB/c nude mice. After 4 weeks, the mice lung tissue was collected for HE staining to observe the presence of metastatic tumors. As shown in Figure 6, the number of metastases significantly decreased with *FGA* overexpression, compared to the control group, indicating *FGA* suppressed HCC cell metastasis formation in the lungs.

## DISCUSSION

In previous studies, FGA has been found to have an effect on tumor metastasis, although its expression levels varied among different cancers. It has been reported that the non-coding sequences between FGA and fibrinogen gamma (FGB) are active in liver cancer cells [32]. Through bioinformatics analysis and agarose gel electrophoresis assay results of liver cancer tissue, we discovered that FGA was significantly downregulated in liver cancer compared to normal liver tissue, suggesting its potential involvement in liver cancer development. However, it remains unclear whether FGA is involved in the invasion and metastasis of liver cancer. To further confirm the function of FGA in liver cancer metastasis, we conducted Transwell migration/invasion assays and wound healing assays, showing that *FGA* overexpression significantly inhibited the migration and invasion of HCC; however, knocking out *FGA* did not alter their migratory or invasive characteristics. Additionally, we established a mouse model for lung metastasis from HCC and performed H/E staining. We observed that FGA had a significant impact on the efficiency of hepatic cell carcinoma metastasis to the lungs. When *FGA* was overexpressed, there was a reduction in visible lung metastatic tumors. The EMT plays a crucial role in the cascade reaction of tumor cell invasion and metastasis. We also confirmed that FGA affected the expression levels of EMT marker proteins through Western blotting and immunofluorescence experiments. Furthermore, we explored possible signaling pathways through which FGA influenced EMT marker protein expression and found that it may be involved in regulating the PI3K/AKT signaling pathway.

When solid tumors undergo hematogenous metastasis, tumor cells enter the circulatory system and interact with coagulation components in the circulation, affecting the formation of cancer cell metastasis. Among these coagulation components, fibrinogen and its cleavage products play a crucial role in metastatic dissemination [33]. Fibrinogen is composed of three peptide chains: FGA, FGB, and fibrinogen gamma (FGG). Different peptide chains have different roles in tumor metastasis. FGB was shown to interact with CD44 to participate in hematogenous metastasis in colorectal cancer [34]. In a clinical observation, it was found that FGB is overexpressed and relatively abundant in urine samples of lung cancer patients, which serve as a biological marker to distinguish between lung cancer patients and healthy individuals [35]. Furthermore, the quantification of FGB and cleaved products may help to further characterize the interconnections between GC and platelet/coagulation pathways [36]. FGG was found to bind various integrin receptors to regulate tumor metastasis in colorectal and breast cancer models. The COOH-terminal globular domain of fibrinogen gamma chain participated in inducing apoptosis of endothelial cells and blocked tube formation of endothelial cells, suppressed tumor growth and metastasis [37]. In a study on anthracycline chemotherapy resistance in breast cancer, it was found that elevated levels of FGG were involved, promoting the survival and proliferation of breast cancer cells [38]. FGA plays a negative regulatory role in tumor metastasis [39]. In a clinical case-control study of breast cancer, FGA levels returned to normal after surgery, showing significant differences compared to preoperative expression [40]. Wang et al. found that knockout of *FGA* induced proliferation and migration of lung adenocarcinoma (LUAD) cells through *in vitro* integrin α5 induction experiments, and induced growth of xenografts and lung metastases *in vivo*, supporting the inhibitory effect of FGA on LUAD cells [41]. In this study, it was also confirmed that FGA has an inhibitory effect on migration and invasion of hepatocellular carcinoma.

The etiology of tumor invasion and metastasis is complex, involving multiple signaling pathways, and the mechanisms of metastasis vary among different types of tumors. Researchers are attempting to find effective measures to restrict the occurrence and development of tumors by continuously studying the mechanisms of tumor metastasis. Tang et al. found that the interaction between LYPLAL1-DT and hnRNPK can restrict the activation of Wnt/β-catenin signaling transduction, thereby exhibiting anti-tumor effects [42], and the biological mechanisms underlying liver metastasis involved circRNAs and immune evasion [43, 44]. The PI3K/AKT signaling pathway is also one of the most commonly activated pathways in tumors. Targeting the PI3K signaling pathway has become a therapeutic strategy to restrict tumor progression [45–48], which participates in a series of signal transduction pathways, including glycolysis, cell apoptosis, autophagy, angiogenesis, and the EMT. Tumor growth and invasion rely on vascular supply, and fine-tuning of the PI3K pathway is essential for tumor neovascularization [49]. In endothelial cells, PI3K promotes angiogenesis through mediation by VEGFR family members and participates in endothelial cell migration. In breast cancer, CYP4Z1 activated tumor angiogenesis and growth through the PI3K/AKT pathway [50]. Interfering with PI3Ka reduced ovarian tumor angiogenesis and growth [51, 52]. The extracellular matrix (ECM) is a complex macromolecular network that tumor cells continuously modify and remodel to acquire invasive and metastatic characteristics [53]. PI3K drives tumor metastasis by promoting cell motility and the EMT [33]. The EMT is coordinated by a group of transcription factors, such as slug, that participate in most steps of the cascade reaction involved in tumor cell invasion and metastasis, enabling tumor cells to detach from their adhesive state into the ECM, leading to increased satellite lesions [54]. Our results corroborated with previous findings regarding the involvement of the PI3K/AKT pathway in liver cancer metastasis. After *FGA* knockout, both p-PI3K and p-AKT expression levels were elevated, indicating potential activation of the PI3K/AKT signaling pathway. Changes in the expression levels of EMT-related proteins, including E-cadherin, N-cadherin, vimentin, etc., can influence the biological behaviors of HCC cells such as proliferation, migration, invasion, and metastasis. This can be helpful in identifying markers that restrict the invasion and metastasis of HCC cells [55, 56]. These changes in expression levels of EMT-related marker proteins in our results suggest promotion of tumor metastasis. Therefore, we infer that FGA participates in liver hepatocellular carcinoma invasion and metastasis through regulation of the PI3K/AKT pathway.

In summary, FGA may inhibit the EMT and subsequently suppress HCC migration and invasion through the PI3K/AKT pathway. FGA has an anti-metastatic effect, and provides a new approach for late-stage HCC treatment.

## MATERIALS AND METHODS

### Differential expression of FGA

We first obtained the pan-cancer expression levels of *FGA* from the TIMER database (https://cistrome.shinyapps.io/timer/) [57], and subsequently, by using the UALCAN database (https://ualcan.path.uab.edu/index.html) [58], we assessed the expression of *FGA* in liver cancer.

### Clinical and survival analysis of FGA in liver cancer

In addition to using online databases for analysis, we downloaded the TCGA-LIHC dataset (https://portal.gdc.cancer.gov/) from the TCGA database [59], which includes 365 tumor samples and 50 normal samples as transcriptional profiles. We analyzed the relationship between *FGA* expression and clinical characteristics of liver cancer by dividing liver cancer patients into high- and low-risk groups using the median expression value of *FGA* as the cutoff, and then exploring whether there were differences in survival between these two groups.

### Molecular network analysis of FGA

We obtained the molecular network of *FGA* from the GeneMANIA database (http://genemania.org/search/homo-sapiens/FGA/) and then calculated the associations between *FGA* and specific molecular pathways using the R programming language (version: 4.30). The results were visualized using the ggplot package.

### Study participants, cell lines, antibodies, and reagents

We have recruited patients and obtained tumor tissue with adjacent normal tissue from 8 patients with HCC who had hepatectomy from December 2022 to March 2023, in a study investigating the role of FGA in the invasion and metastasis of HCC. Only those who consented, or whose legally authorized representative consented, were enlisted in the study. Written informed consent was obtained from all participants. All of the specimens were confirmed by pathological diagnosis and approved for use by the ethics committee of the Second Hospital of Jilin University (2021-172). The human hepatocellular carcinoma cell lines used in this study included HL-7702, Hep3B, Huh7, HepG2, and HCC-LM3, among which HL-7702, Hep3B, Huh7 and HepG2 were obtained from Cell Bank, Chinese Academy of Sciences, and HCC-LM3 was obtained from Zhongshan Hospital of Fudan University. Cells were cultured in an incubator at 37° C with 5% CO_2_. Antibodies were purchased from Cell Signaling Technology (Danvers, MA, USA). RIPA cell lysis buffer and a Bradford protein concentration assay kit were obtained from Beyotime (Shanghai, China).

### Establishment of the FGA knockout/overexpression liver cancer cell model

We used the CRISPR online design tool to design paired sgRNAs sequences targeting *FGA* (from Benchling, San Francisco, CA, USA, https://benchling.com). In order to avoid off-target effects, we utilized the Cas-OFFinder tool (from Daejeon, South Korea, http://www.rgenome.net/cas-offinder) to identify potential off-target regions, which were then validated by PCR and Sanger DNA sequencing. Subsequently, the designed sgRNA sequences targeting *FGA* were incorporated into the pSpCas9(BB)-2A-GFP (PX458) vector that had been digested by the BbsI restriction enzyme (sourced from Addgene, Cambridge, MA, USA). After that, the recombinant vector was transfected into HepG2 cells. With GFP as a selection marker, we used flow cytometry (BD FACSMelody™ Cell Sorter, Franklin Lakes, NJ, USA) to screen for single-cell clones. Single clones with reduced FGA expression were then selected based on real-time quantitative PCR and protein immunoblotting techniques for subsequent experiments.

In the process of establishing the *FGA* overexpression cell line, we used site-directed mutagenesis techniques. We simulated these gene mutations in the *FGA* overexpression plasmid. We then transfected the recombinant plasmid into HepG2 cells where *FGA* had been knocked out. Through drug screening, we obtained a stable HepG2 cell model with *FGA* overexpression.

Target gene primer

**Table:** 

*FGA* (72215-2)-p1	GTGGATCCGAGCTCGGTACCCGCCACCATGTTTTCCATGAGGATCGTC
*FGA* (72215-2)-p2	ATATTTTATTACCGGTTTAATTAACTAGGGGGACAGGGAAGGCTTCC

Recombinant plasmid primer

**Table:** 

*FGA* (72215-2)-p3	GGCCAGATAGCCCAGGCTCT
*FGA* (72215-2)-p4	GAGCGTATGTTAGTACTATCG

### Quantitative real-time PCR

Total RNA of tissue samples and cell lines was attained with Trizol reagent (Invitrogen, Carlsbad, CA, USA) under guidelines of the manufacturer. Reverse transcription was performed using a Quanshijin reagent kit (Beijing Quanshijin Biotechnology Co., Ltd., China) to synthesize cDNA. Experiments were conducted using a fluorescence quantitative PCR instrument (Bio-Rad, Hercules, CA, USA). Quantitative analysis was carried out using software provided by Bio-Rad. FGA primers: Forward 5’-GGACAATGGCACTCTGAATCT-3’; Reverse 5'-GTGACCATCAGGACCAATAACA-3’.

### Agarose gel electrophoresis assay

The 50× TAE buffer was prepared with 242 g of tris base (provided by Suzhou Chemical Technology Co., Ltd., China), 57.1 mL of acetic acid, and 0.1 L of 0.5 mol/L EDTA, diluted to 1 L. For preparing the agarose gel, an appropriate amount of agarose was added to 100 mL of 1× TAE electrophoresis buffer. Ethidium bromide was added, mixed well. Then, 500 mg of agarose in 50 mL of TAE was boiled, and a trace amount of ethidium bromide was added to prepare the gel. On the ultra-clean workbench, aspirated 4 μl of total RNA sample using a pipette, then added 5 μl of 1× TAE electrophoresis buffer and 1 μl of 10× loading buffer on the bench. Mixed well and carefully loaded into the sample wells. Adjusted the voltage to 100 V for RNA electrophoresis, with the RNA migrating from the negative electrode to the positive electrode. After approximately 30 minutes, transferred the gel into EB staining solution and stained for 5 minutes, followed by a brief rinse with water. Observed the RNA electrophoresis results on a UV transilluminator.

### Transwell migration/invasion assay

Added DMEM complete culture medium containing 10% FBS to the lower chamber, and serum-free culture medium to the upper chamber, followed by the addition of resuspended cells without FBS. The setup was incubated for 24 hours. After, the culture medium was discarded, paraformaldehyde was added and fixed at room temperature. The cells were then stained for 5 minutes, rinsed with PBS buffer three times, and left to air-dry at room temperature. Photos were taken using an inverted microscope, and cells were counted in five random fields of view.

### Wound healing assay

Cells after *FGA* knockout were seeded in a six-well plate. A sterile 10-μL pipette tip was used to make a scratch vertically to the horizontal line in each well, then rinsed with PBS three times. The scratches were photographed using an inverted microscope and the width of the scratches was recorded. The cells continued incubation, and photos were taken in the same position after 12 hours and 24 hours.

### Western blotting

Briefly, proteins were extracted from the collected cells by RIPA buffer, analyzed using SDS-PAGE and electro-transferred to a PVDF membrane. After blocking, the membrane was incubated with the primary antibody overnight at 4° C. The next day, the membrane was incubated with the secondary antibody for 1 hour. β-Actin was used as the internal reference protein. Membranes were visualized and analyzed using an ECL chemiluminescent substrate and an ECL imaging system.

### Immunofluorescence

HepG2 cells (wild type and overexpressing FGA) were seeded in a 24-well plate. Cells were fixed with a 4% paraformaldehyde solution (Beyotime Biotechnology, P0099) for 25 minutes, treated with 20–30 μg/mL proteinase K for 1 minute, blocked with 5% goat serum (prepared in 0.01 M PBS) in a humid box at room temperature. Then, cells were incubated with the primary antibody. Subsequently, cells were placed in the dark at room temperature in a humid box for 30 minutes, treated with an appropriate volume of 1 μL/mL Hoechst 33342 dye. The slides were then sealed using an aqueous mounting medium and images were captured using a laser confocal microscope.

### Hematoxylin and eosin staining (H/E staining)

Lung tissue was fixed with paraformaldehyde and then processed into paraffin sections. Before observation, the sections were dewaxed, rehydrated, stained with hematoxylin and eosin, dehydrated, cleared with xylene, and then mounted and allowed to dry.

### *In vivo* tumor metastasis assay

Wild-type, *FGA* knockout, and *FGA* overexpression HepG2 cells were resuspended in a 0.9% saline solution. The mice (4 weeks of age, immunodeficient BALB/c nude mice) were injected with 3.0 × 10 ^ 5 cells in the liver. The mice were euthanized after 4 weeks, and their lung lobes were analyzed with hematoxylin and eosin (H/E) staining.

### Statistical analyses

The data were statistically analyzed using SPSS 22.0 and GraphPad Prism 8 software. The survival and clinical analysis related to FGA were conducted using the R software (version 4.2.1) with the limma, survival, and survminer packages. The molecular network plot depicting FGA and its related pathways was generated using the corrplot package. The graphical representations were supported by the ggpubr and ggplot2 packages. The log-rank test was employed in Kaplan–Meier curve to compare the survival rates between groups. Independent sample Student’s t test was exploited to contrast the expression of FGA between tumor tissues and nontumoral tissues. Comparisons were implemented by dint of the two-tailed Student’s t test between two groups and one-way analysis of variance (ANOVA) test with Bonferroni correction among three groups. All experiments were executed at least in triplicate. For bar plots, data were shown as the mean ± SD. The statistical significance notation is as follows: “ns” indicates no significant difference; *p indicates p < 0.05; **p indicates p < 0.01; and ***p indicates p < 0.001.

### Data availability statement

The raw data supporting the conclusions of this article will be made available by the authors, without undue reservation.
